# Promoting cardiovascular health and wellness among African-Americans: Community participatory approach to design an innovative mobile-health intervention

**DOI:** 10.1371/journal.pone.0218724

**Published:** 2019-08-20

**Authors:** LaPrincess C. Brewer, Sharonne N. Hayes, Amber R. Caron, David A. Derby, Nicholas S. Breutzman, Amy Wicks, Jeyakumar Raman, Christina M. Smith, Karen S. Schaepe, Ruth E. Sheets, Sarah M. Jenkins, Kandace A. Lackore, Jacqueline Johnson, Clarence Jones, Carmen Radecki Breitkopf, Lisa A. Cooper, Christi A. Patten

**Affiliations:** 1 Department of Cardiovascular Medicine, Mayo Clinic, Rochester, Minnesota, United States of America; 2 Robert D. and Patricia E. Kern Center for the Science of Health Care Delivery, Mayo Clinic, Rochester, Minnesota, United States of America; 3 Division of Health Care Policy and Research, Mayo Clinic, Rochester, Minnesota, United States of America; 4 Department of Research Administration, Mayo Clinic, Rochester, Minnesota, United States of America; 5 Division of Biomedical Statistics and Informatics, Mayo Clinic, Rochester, Minnesota, United States of America; 6 Christ’s Church of the Jesus Hour, Rochester, Minnesota, United States of America; 7 Hue-MAN Partnership, Minneapolis, Minnesota, United States of America; 8 Department of Medicine, Johns Hopkins School of Medicine, Baltimore, Maryland, United States of America; 9 Department of Health, Behavior and Society, Johns Hopkins Bloomberg School of Public Health, Baltimore, Maryland, United States of America; 10 Department of Psychiatry and Psychology, Mayo Clinic, Rochester, Minnesota, United States of America; Pennington Biomedical Research Center, UNITED STATES

## Abstract

**Background:**

Despite improvements in mortality rates over the past several decades, cardiovascular (CV) disease remains the leading cause of death for African-Americans (AAs). Innovative approaches through mobile health (mHealth) interventions have the potential to support lifestyle change for CV disease prevention among AAs. We aimed to translate a behavioral theory–informed, evidence-based, face-to-face health education program into an mHealth lifestyle intervention for AAs. We describe the design and development of a culturally relevant, CV health and wellness digital application (app) and pilot testing using a community-based participatory research (CBPR) approach with AA churches.

**Methods:**

This mixed methods study used a 4-phase iterative development process for intervention design with the AA community. Phase 1 included focus groups with AA community members and church partners (n = 23) to gain insight regarding potential app end user preferences. In Phase 2, the interdisciplinary research team synthesized Phase 1 input for preliminary app design and content development. Phase 3 consisted of a sequential 3-meeting series with church partners (n = 13) for iterative app prototyping (assessment, cultural tailoring, final review). Phase 4, a single group pilot study among AA church congregants (n = 50), assessed app acceptability, usability, and satisfaction.

**Results:**

Phase 1 focus groups indicated general and health-related apps preferences: multifunctional, high-quality graphics/visuals, evidence-based, yet simple health information and social networking capability. Phase 2 integrated these preferences into the preliminary app prototype. Phase 3 feedback was used to refine the app prototype for pilot testing. Phase 4 pilot testing indicated high app acceptability, usability, and satisfaction.

**Conclusions:**

This study illustrates integration of formative and CBPR approaches to design a culturally relevant, mHealth lifestyle intervention to address CV health disparities among AAs. Given the positive app perceptions, our study supports the use of an iterative development process by others interested in implementing an mHealth lifestyle intervention for racial/ethnic minority communities.

**Trial registration:**

Clinicaltrials.gov NCT03084822.

## Background

Cardiovascular (CV) disease affects nearly half of all African-American (AA) adults, who have a 30% higher age-adjusted death rate from CV disease than the overall population in the United States [1–3]. Approximately three-fourths of absolute disparities between AAs and whites in CV disease mortality may be attributable to differences in multiple traditional CV risk factors, including hypertension, diabetes mellitus, obesity, physical inactivity, and poor diet [4]. As in other areas of the United States, AAs in Minnesota have higher rates of uncontrolled CV risk factors, leading to higher premature mortality rates from CV disease than those of the predominantly white state population [5–8]. Many of these CV disease–related deaths could be avoided through improvements in modifiable lifestyle behaviors and risk factors and by addressing the social determinants of health (ie, socioeconomic and cultural factors that influence the health of individuals and communities) [9]. Culturally tailored preventive efforts and interventions to mitigate CV health disparities within this population are critically needed because of compelling evidence suggesting that AAs are at higher risk for CV disease than they actually perceive [10–12].

Community-based participatory research (CBPR) is a type of community engagement in which academic and community partners collaborate in the conceptualization and design of interventions to address health disparities [13]. CBPR can be used to engage a prioritized population to increase cultural relevance, acceptability, and sustained use of interventions and has been successful in increasing awareness of CV risk factors and in promoting healthy lifestyle among AAs [14–16]. Partnerships with the AA faith community have been particularly effective in improving the CV health of AAs [17, 18]. In our own face-to-face CV disease prevention program (FAITH! [Fostering African-American Improvement in Total Health]), we utilized a CBPR approach with a faith-based organization to positively impact CV health literacy and risk factors of AA churchgoers [19]. Shared insights from the academic-community partnership revealed a strong need to increase accessibility of CV health education to a broader AA community through a novel approach of integrating mobile technology [20].

A growing body of evidence supports the use of mobile health (mHealth) interventions, defined as “medical and public health practice supported by mobile devices such as mobile phones, patient monitoring devices, personal digital assistants, and other wireless devices,” [21, 22] that are used for health promotion, behavior change, diagnosis, and self-management of risk factors and medical conditions [23]. The ubiquity of mobile phones, with few differences in use between whites and AAs across demographic categories, shows the potential of mHealth interventions for addressing CV health disparities of racial/ethnic minority groups [22, 24].

The use of mHealth interventions is increasing substantially in the general population, and it is important to ensure that CV health disparities do not widen in underserved communities from lack of evidence-based, culturally relevant interventions. A recent statement from the American Heart Association highlighted the need for innovative changes in mHealth interventions designed to incorporate evidence-based content with rigorous testing in more diverse populations, including AAs [25]. However, only a few studies have sought to address this need by using CBPR to test the feasibility of culturally tailored, mHealth lifestyle interventions among this high-risk population [26–29]. The use of mobile technologies, such as text messaging and physical activity monitoring devices, to address hypertension and obesity disparities in the AA community appears feasible and acceptable [26, 27]. Thus, mHealth lifestyle interventions, which are relatively low in cost and easily adaptable, could substantially impact the health of racial/ethnic minority communities, which are often medically underserved, disadvantaged, and underresourced [30–32].

Ongoing community input within our established academic-community partnership with AA church congregations led to the joint decision to design and develop a digital application (app) coined FAITH! App, which is focused on CV health and wellness among AAs. Guided by feedback from our community partners and key stakeholders within the AA community, we translated a behavioral theory–informed, evidence-based, face-to-face health education program to a multimedia, interactive, on-demand mHealth lifestyle intervention. The purpose of the current paper is to describe the iterative process of design and development of the FAITH! App prototype through a mixed-methods CBPR approach and to provide the initial results of a pilot study assessing its acceptability, usability, and user satisfaction.

## Methods

The study was approved by the Mayo Clinic Institutional Review Board and registered on clinicaltrials.gov (NCT03084822).

### Setting and CBPR partnership

The FAITH! Program is a behavioral theory–informed, culturally tailored, community-based CV health and wellness program implemented as an academic-community partnership with our institution and local AA churches in Rochester, Minnesota [33]. The program’s community-identified and overarching mission is to address CV health disparities among AAs in Minnesota through innovative lifestyle interventions. CBPR principles have guided the ongoing FAITH! programming and study design. The established FAITH! Program community partners (church partners consisting of 3 church congregations) and study team set a goal to develop an mHealth lifestyle intervention, which was based on review of prior face-to-face intervention program evaluations [20, 33]. Furthermore, the church partners and study team mutually decided to expand to more church congregations in Rochester and the Minneapolis-St. Paul area to broaden the program’s reach. To facilitate this goal, the study principal investigator (L.C.B.) established a new relationship and partnership with the community outreach director (C.J.) of a trusted, federally qualified health center in Minneapolis-St. Paul, grounded in collaborations with AA church congregations. Three new church congregations (1 in Rochester and 2 in Minneapolis-St. Paul) were selected for their positive reputations in the AA community and interests in community health promotion. Following an introductory, in-person meeting with the study team to discuss potential collaboration in the expanded program with leadership representatives of each church (pastors and auxiliary leaders), all 3 churches expressed formal interest in partnering with the FAITH! Program. The church partners advised translating the face-to-face FAITH! Program to an mHealth digital app lifestyle intervention to be less resource-intensive and easier to disseminate to the AA community.

### Conceptual framework

The current intervention and educational content was informed by a conceptual framework inclusive of behavioral theories previously integrated into the face-to-face FAITH! Program [19], including tenets from the health-belief model [22], social cognitive theory [34], and the community mobilization model [35, 36]. In translating the face-to-face program to an mHealth intervention, we prioritized these theories as a foundation to increase the likelihood of its effectiveness [37]. The health-belief model, which incorporates an individual’s perceived susceptibility, benefits, and barriers to predict behavior change, directed development of moderator guides for the focus groups and app content (ie, self-assessments). Social cognitive theory, or learning through a social context, informed our inclusion of a discussion sharing board and testimonials on healthy lifestyle from AA community members in recognition of the interpersonal influences of behavior change. The central focus remained on providing support for behavior change (healthier diet and increased physical activity) among AAs. Through the community mobilization model, our community members were mobilized to address a community health issue (CV health disparities in the AA community) and support positive health outcomes for the prioritized population. Church partners emphasized the necessity of involving the AA community in creating, endorsing, and implementing the intervention components to ensure its relevance (attention to norms, values, and needs) and to allow for equitable sharing of power, strengths, and resources.

### Overview: Study phases

**Fig 1:**
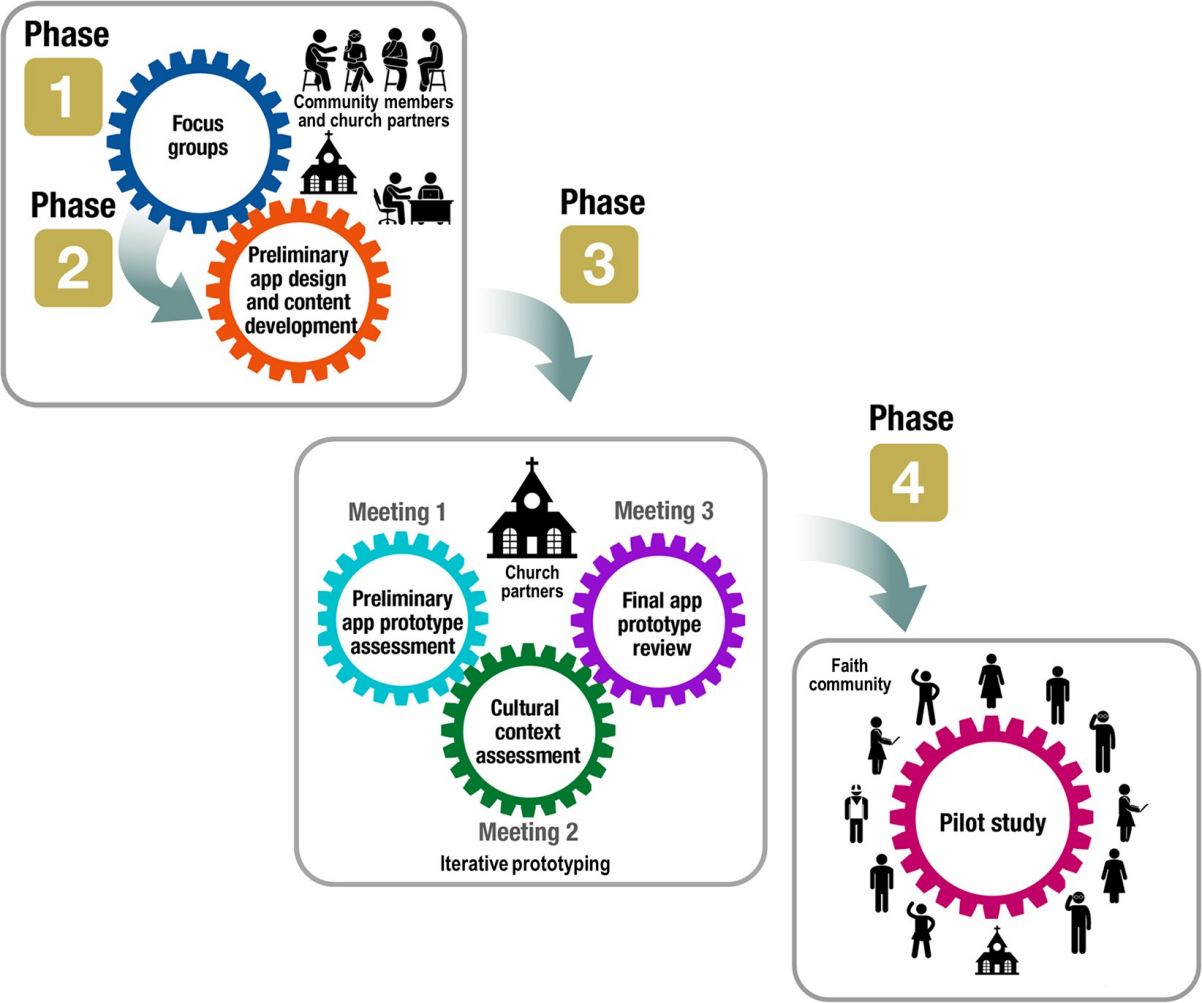
Phases of FAITH! App design, development, and pilot testing. FAITH! indicates Fostering African-American Improvement in Total Health.

A series of meetings were held with the study team and church partners to outline 4 sequential phases for development of the mHealth intervention and subsequent pilot study, as adapted from prior behavioral interventions [38–42]. The interdisciplinary research team included expertise in CV medicine (L.C.B., S.N.H.); general internal medicine (L.A.C.); psychology (C.A.P., C.R.B.); CBPR (C.A.P., C.J., J.J., L.C.B., L.A.C.); biostatistics (K.L., S.J.); qualitative methods (C.M.S., K.S.S.); health literacy, digital communication, and electronic learning (R.E.S.); graphic design (N.S.B.); computer programming (J.R.); and software development, information technology (J.R.), and program management (A.R.C., A.W., D.A.D.). The key phases are displayed in Fig 1.

### Phase 1: Focus groups

#### Procedures

The rationale for this phase was to incorporate views of potential end users into the intervention design to maximize user acceptability and satisfaction with the intervention. We also sought to build rapport, trust, and credibility with our prioritized audience, the AA community. Exploring the wants and needs for app design in this manner was important because a group environment naturally stimulates idea exchange and allows for real-life social contextual understanding and cultural influences [43]. We used purposeful sampling [44] to engage with 23 AA participants (16 community members and 7 church partners) for 2 focus groups with complementary but distinct objectives. Following review of the study objectives and timeline of the focus group series, verbal informed consent was obtained, and a $50 participation incentive was provided. Participants completed a 17-item written survey probing sociodemographics, mobile technology use patterns, sources of health information, and interest in learning more about healthy behaviors and CV disease prevention. Each session took place at a convenient venue (eg, community clinic) on weeknights, ranged from 90 to 120 minutes, and was audio recorded. Before each session, participants shared a meal and informally discussed community health and events. All meetings began with an opening prayer by church leadership to set an atmosphere of creativeness, inspiration, and togetherness among the attendees.

### Focus group 1: Community members

Through church announcements, flyers, and email contact, we recruited a sample of AA adults from Rochester and Minneapolis-St. Paul churches, community health centers, schools, and the Mayo Clinic African Descendants Employee Resource Group who had not participated in the face-to-face FAITH! Program. Our aim was to obtain a variety of viewpoints and input from AAs, both within and outside the faith community, regarding mobile technology use patterns, preferences about general and health-related apps, and receptivity to mHealth interventions for AAs. Participants were asked questions about daily tasks they completed using mobile technologies, disengaging/engaging features of digital apps (general and health related), and their overall interest in learning more about improving CV health. In addition, we introduced the preliminary basic app functions by displaying color screen shots and handouts for the proposed home page, education modules, and self-monitoring tracking function.

### Focus group 2: Church partners

We met with 13 established and new church partners (including church pastors and former face-to-face FAITH! Program participants) to discuss patterns of mobile technology use and app preferences as well as to review draft images of the proposed app home page and basic functions. Our objective was to obtain additional input on essentials to retain from the face-to-face FAITH! Program. Participants also viewed and provided feedback on samples of education module videos, the proposed core of the health education component of the intervention.

#### Analysis

The study team created moderator guides with topics focused on mobile technology use and proposed app features. One study team moderator (C.M.S.) facilitated the conversation, and a rotating co-moderator served as a note taker (A.W., D.A.D., or N.S.B.), attending to not only comments made during the session but also to gestures, eye contact, general demeanor, and other nonverbal behavior. Immediately after each meeting, a list of topics discussed during the session was compiled by the moderators. This included summarizing the range of comments into theme-based codes, while conferring about special in vivo terms [45] used by participants that might merit inclusion in a subset of codes. Furthermore, this included a preliminary comparison of participant views within and between different focus group sessions. The audio recordings were transcribed verbatim, and the set of transcripts was sent to be reviewed by an analyst independent of the project (K.S.S.). Incorporating an independent analyst in this manner offered a second impression or interpretation of the group discussions. We purposefully built this step into the analysis design to provide confirmation of central themes previously identified by the moderators and as a protective measure to help offset biases and preconceptions of the primary study team members.

The outside analyst utilized a two-step review process: First, a descriptive content analysis was conducted. This involved tallying and then organizing the discussion of key issues previously identified by the study team regarding user experience (eg, discussion of ease or difficulty in reading text and mention of preferences for style of notifications). Summary of this content analysis was directly cross-referenced to the ad hoc theme-based coding completed by the moderators following the focus group sessions. Second, an interpretive analysis was performed that was informed by symbolic interactionism, which is a conceptual framework that foregrounds reciprocal interaction, a way that individuals adapt to their social environment [46, 47]. The analysis process involved the analyst reading the transcripts while concurrently listening to the audio recordings to develop a better sense of affect reflected in nontextual features of the discussion (eg, tone, prosody, pauses, and sighs). The analyst coded less explicitly identified patterns in participant comments regarding the ability and willingness to adapt to a novel mHealth lifestyle intervention and concerns relevant to the faith community context. This review process yielded a number of major themes regarding mobile technology use and the proposed intervention. The analyst then presented the compilation of major themes to the study team for review, which was utilized in Phase 2 for preliminary app prototyping and content development (as detailed below). Subsequently, illustrative quotes were selected that corresponded to major themes and participant feedback.

### Phase 2: Preliminary app prototype design and content development

#### Procedures

The interdisciplinary team worked with software developers to synthesize and triangulate data from Phase 1 community input and the literature [22, 48–52] to outline basic app features while incorporating behavioral theories from our conceptual framework. Our goal was to develop a preliminary app prototype for review and approval by the church partners. For each app function/feature, we collaborated with a graphic designer and software developers to draft a graphic and infrastructure design by using navigation flow charts to display end-user paths for each app page.

#### Analysis

Given time and financial constraints and our need to maintain accountability with the church partners, we used the prioritization model MoSCoW (Must have, Should have, Could have, Would like) [53] to consolidate the basic functions/features of the app we envisioned while ensuring its cultural sensitivity, usefulness, and user satisfaction for promoting CV health among the AA faith community.

### Phase 3: Iterative prototyping

#### Procedures

During this phase, 3 meetings were held with the Phase 1, Focus Group 2 church partners for sequential design of the app prototype [54].

Meeting 1: Preliminary App Prototype Assessment. Participants were given a preview of the semifunctional app prototype developed in Phase 2. As outlined by Bradbury and colleagues [41], the meeting was structured as a “think aloud” session to assess the partners’ initial perceptions of the app. Participants were further encouraged to express how the app could better meet the needs of the AA community in fostering healthy lifestyle change. Each participant tested the usability of the app features and navigation on a tablet device with a self-guided script. Participants were asked to complete certain tasks, including the login process and each basic app feature. Study team members monitored their actions, reactions, and any difficulties, which allowed the team to identify any technical glitches the software programmers needed to resolve.

Meeting 2: Cultural Context Assessment. The next meeting focused on positioning the proposed mHealth intervention into the cultural context of potential participants from the AA faith community along with an assessment of group expectations for the proposed app prototype pilot study. The study team also elicited feedback on the culturally tailored features of the intervention. The principal investigator reviewed a timeline that included logistics of the recruitment strategy, outcome measures, data collection methods, implementation plan, and long-term goals for disseminating the mHealth intervention to the larger AA community.

Meeting 3: Final App Prototype Review. The third meeting was a final review by the church partners of all revised app components that was based on insights, feedback, suggestions, and analyses from meetings 1 and 2. The study team also elicited feedback on the process of involving the AA faith community in app development.

#### Analysis

Similar qualitative methods were followed as in Phase 1. Moderator guides were developed sequentially on the basis of participant feedback regarding the app prototype design at each meeting.

### Phase 4: Pilot study

#### Procedures

Next, the team returned to the overall project objective of pilot testing the FAITH! App within the priority population, with the goal of ensuring that it was consistent with their preferences, generally usable, and useful for learning about CV health. Fifty participants were recruited from 5 predominantly AA church congregations within Rochester (n = 24) and Minneapolis-St. Paul (n = 26) through a CBPR approach [33]. Inclusion criteria for participants were the following: AA, age at least 18 years, basic Internet use skills (navigating, searching for information, or downloading files) [55], at least weekly Internet access, active email address, minimal fruit/vegetable intake (<5 servings/day), no regular physical activity program (<30 minutes/day of moderate physical activity), and able to engage in moderate physical activity (no restrictions). Individuals were ineligible if they were pregnant, had visual or hearing impairment, had mental disability that would preclude independent use of the app, or were former participants of either the face-to-face FAITH! Program [19] or Phase 3 iterative app prototyping. After informed consent was obtained, those included in the study were given a tablet device equipped with the FAITH! App to use throughout the study. They also received a $50 participation incentive at baseline and at completion of the intervention. To enhance intervention engagement and study retention, at least one FAITH! Partner was designated from each participating church to serve as a key contact person should participants have issues with the app. The FAITH! Partner also encouraged participants by delivering standardized church announcements prepared by the study team. This tactic was successfully utilized in the prior face-to-face FAITH! intervention [19]. Participants completed baseline, postintervention (at 10 weeks), and final (at 28 weeks) electronic surveys that assessed sociodemographic characteristics and user perceptions of app acceptability, usability, and satisfaction. Within the single-group, nonrandomized pilot study, participants followed a 10-week intervention phase based on the education module topics.

#### Analysis

To assess acceptability, participants were asked to rate the FAITH! App as follows: 1) degree met needs for gaining knowledge and skills to make healthy lifestyle choices (scale, 1 to 5: 1 not at all well to extremely well), 2) likelihood of recommending to a family member, friend, or colleague (scale, 0 to 5: not at all likely to extremely likely), and 3) overall rating (scale, 1 to 10: poor to excellent). Usability was assessed by an adapted version of the Health Information Technology Usability Evaluation Scale (Health-ITUES), a customizable mHealth-intervention–usability assessment instrument previously validated among community-dwelling adults using a chronic disease–related app [56]. The Health-ITUES comprises 20 items grouped by 4 subscales: 1) impact, 2) perceived usefulness, 3) perceived ease of use, and 4) user control. Each item is rated on a 5-point scale from 1, strongly disagree to 5, strongly agree—higher scale values indicating greater perceived usability of the mHealth intervention. We selected 9 items from the Health-ITUES that were most relevant and least redundant for each subscale and adapted them for use in evaluating the FAITH! App: 1) impact (2 items: FAITH! App positive addition to church and community; FAITH! App important part of our church health ministry); 2) usefulness (1 item: FAITH! App useful for learning about heart health); 3) perceived ease of use (4 items: FAITH! App easy to use; comfortable with my ability to use the FAITH! App; easy to become skillful at using the FAITH! App; received sufficient education to log on and use the FAITH! App); and 4) user control (2 items: whenever make a mistake using FAITH! App, recover easily and quickly; clear information [online help, on-screen messaging, other documentation] provided). An overall Health-ITUES score was calculated as the mean of all 9 items, with each item equally weighted. Subscale scores were similarly calculated as the mean of the items within each subscale. Each of the acceptability and usability outcomes was summarized as mean and standard deviation (SD) or interquartile range (IQR), as appropriate. Each of the app features (eg, education modules, tracking function, sharing board) was evaluated for user satisfaction (very dissatisfied to very satisfied). Each satisfaction outcome was summarized as frequency (percentage) with satisfied and very satisfied ratings combined.

## Results

### Phase 1: Focus groups

#### Participants

The AA community members and church partners had the following sociodemographic characteristics: women, 74%; mean age (SD, range), 55 (11.8; 31–72) years; at least college graduate, 65%; annual household income <$50,000, 30%; high mobile technology use (smartphone ownership, 83%; ≥4 hours of use/day, 86%); comfortable with mobile technology, 74%; and interested in learning more about healthy behaviors and CV disease prevention, 100%.

#### Findings

Participants in both focus groups expressed an overall interest in integrating mobile technologies inclusive of digital apps into their daily lives. They indicated active use of general apps for a wide range of purposes encompassing social media, music, and driving navigation assistance (eg, Facebook, Instagram, Snapchat, Pandora, YouTube, Shazam, Google Maps). Their health-related app use included those from commercial entities (eg, Fitbit, MyFitness Pal), government agencies (eg, MyPlate), and several academic medical centers.

Table 1 summarizes the key themes and subthemes (collective feedback and preferences for general and health-related digital apps) provided by the participants within both focus groups. For general apps, they preferred those that were multifunctional and that could be personalized to meet their specific needs: “to be able to customize for whoever is using it.” Visually appealing apps, whether general or health-related, were more frequently visited: “If I have a picture to help me out, I’ll come back.” In terms of health-related apps, most participants stressed the importance of receiving health information from a reputable and trusted source: “a belief that the information is accurate,” “it’s a valid source.” Participants wanted health-related apps to have specific features to help them maintain health behaviors, including notifications/reminders, goal setting, challenges, and self-assessments: “accomplishing goals or tasks or something you can check off so you feel like you’ve done something to be healthier,” “something that you can quiz yourself…and then have the results of how you add up those questions…to help you get on the right track or maybe pat yourself on the back.” They also felt much more motivated to engage with health-related apps that fostered social engagement and spirituality/health connections: “the spiritual ties between taking care of our temple and taking care of our physical health. So the spiritual connection to mind-body wellness.” Unfavorable aspects of both general and health-related apps were overwhelming text, challenging navigation, and a steep learning curve for basic use: “Sometimes you can get too much information, then you really become more confused…overwhelmed. I don’t know what to do with it.”

**Table 1:** Phase 1: Preferences of community members and church partners for general and health promotion digital apps (n = 23).

Themes	Subthemes	Selected Illustrative Quotes
**Engaging features**		
General apps		
Multifunctional	Tracking of desired behavior	“I think when an app is exciting and colorful, you know. And descriptive…all those things so that it just pops, you know. I think that’s when, when you want to share information with other people.” “But my personal preferences are drawn towards more of photographs. …potentially of people.”
	Goal setting	
	Interactive features to increase engagement	
Personalization/customization	Recognition as a unique user	
	Tailored to personal interests	
Visuals	Videos, pictures, and photographs to provide instructions and increase use	
Health-related apps		
Accurate, trusted health information	Simple, minimal medical jargon	“I think it’s being simple enough so that everybody can understand it. ‘Cause some things, some people may understand and some may not. So if it’s simple enough for all age groups, backgrounds…” “When we challenge people—have friends and you challenge them. A challenge week, and you kinda do a little extra. You wanna win the challenge.” “…the spiritual ties between taking care of our temple and taking care of our physical health. So the spiritual connection to mind-body wellness.”
	Variety of topics	
Visuals	Videos and photographs of health conditions and diseases	
Positive reinforcement for healthy behaviors	Notifications/reminders	
	Goal setting	
	Challenge	
	Self-assessments	
Concept of social engagement	Create sense of community	
	Welcoming environment	
	Ability to share information with others	
Concept of physical and spiritual well- being	Spiritually infused content	
	Physical and spiritual health connection	
**Undesirable features**		
General		“You get to reading it and it just goes on and on and on, and you decide no. I don’t wanna—I’m not gonna read all this.” “Sometimes a lot of stuff will come up, and then…you click on it and you have to click on something else, and then you still don’t have what you need.”
Dense text		
Difficult navigation		
Health-related apps		
Heavy medical jargon		
High learning curve		
Requirement to search for information		

Abbreviation: apps, applications.

There was a consensus among the second focus group participants in favor of preserving high-quality CV health education from health professionals as the core of the mHealth intervention as a translation of the face-to-face health education program to the app. The group indicated strong value of the group learning environment from the face-to-face program, which was conducive to sharing experiences, asking questions, and retaining information: “During our pilot (FAITH! face-to-face program), we were there together. We heard it together. We sat down, we ate together, we sat down and we listened to the physicians together. We talked together. That’s the part I’m saying is different.” They encouraged the study team to include a few optional live events for all congregations to discuss health promotion to maintain a sense of cohesiveness. They specifically mentioned the usefulness of the cooking demonstration and cookbook from the prior FAITH! intervention [19] for participants to learn new and heart-healthy ways to prepare dishes/meals and to share and ask questions of each other and the study team: “…we have special foods as African-Americans—our soul food…it was very interesting how the chef prepared the soul food which tasted like soul food, but it wasn’t cooked like we normally cooked it. So the cooking classes, the recipes that we got in the cookbook—all of that was excellent.”

### Phase 2: Preliminary app prototype design and content development

#### Findings

Each app feature and its systematic development process is described below and further detailed in Table 2 [34, 57–69]. We provide insight into our synthesis of community input from Phase 1, available evidence on effective and user-acceptable mHealth intervention components, and behavior theory models toward the intervention development. Given the paucity of prior mHealth interventions specifically tailored to AAs, integration of the community’s identified preferences and expectations were of crucial priority in our app development efforts.

**Table 2:** Basic functions of the FAITH! App.

Function	Description	Rationale for Inclusion (Community Input, Evidence-based, Behavioral Theory)
Education modules	Core video series on key CV risk factors, health behaviors, and CV health promotion from health professionals; pre- and post-module self-assessments and relevant brochure content included in each module	Community input: Phase 1 participants indicated need to provide accurate, trusted health education to promote CV health. Evidence base: Health professional involvement increases user confidence in app content and can increase usage [57]. A need remains for CV health education among African-Americans (particularly women), as they are more likely to underestimate their CV risk because they lack knowledge of the risk factors associated with CV disease [58–60]. Behavioral theory: Fundamental to the Health Belief Model construct is the notion that individuals who perceive more benefits than barriers to healthy behavior change are more likely to take action [61]. Cues to action are means to change these perceptions and to promote CV health.
Self-monitoring/tracking	Interactive tracking of fruit and vegetable intake and physical activity via a monthly calendar	Community input: Tracking was viewed as a means to increase app engagement and personal accountability for behavior change. Evidence base: A systematic review showed the value of self-monitoring features in mobile app behavior change interventions [62, 63]. Apps including performance measurements and self-monitoring showed an association with greater effectiveness [64]. Behavioral theory: The Health Belief Model was leveraged within the education modules to increase the perceived benefit of healthy eating and regular physical activity in reducing risk of CV disease, thus increasing the likelihood that participants would take action and engage in these health behaviors [65].
Sharing board	Discussion platform and feed for participants to post healthy lifestyle practices and interact with each other	Community input: Phase 1 expressed a need for opportunities to share questions and experiences for healthy lifestyle change. Fostering camaraderie and social connectedness was a key preference. Evidence base: Systematic reviews have shown the value of creating a sense of relatedness [62, 63] and social networking through peer support forums in health behavior interventions [64]. Behavioral theory: Employing the Social Cognitive Theory [34] can increase self- efficacy in one’s ability to perform a behavior by observing another individual perform the behavior successfully. Individuals are more likely to model this behavior if they identify with the individual. Also, this theory provides social support reinforcement for changes in behavior.
Testimonials	Church leadership and former FAITH! Program participant video accounts of personal experiences with the program, as well as motivational messaging	Community input: The church partners (in tandem with the study team) wanted an avenue to include viewpoints from prior participants of the FAITH! Program to increase its credibility and relatability to the African-American community. Evidence base: There are several studies including testimonials, first-person narratives, or anecdotes to communicate health risk and to persuade individuals to engage in particular health behaviors, preventive measures, and decision making [66]. Behavioral theory: An integration of both the Health Belief Model and Social Cognitive Theory can promote self-efficacy through motivational messaging and observation of others [67].
Recipes	Cookbooks, including heart-healthy meals focusing on traditional African-American cuisine	Community input: Participants previously found culturally tailored recipes appealing to accomplish the goal of healthy eating. Participants encouraged active cultural humility by the study team through reflection on and acknowledgement of the uniqueness of the African-American community’s traditions, personal beliefs, and preferences [69]. Evidence base: A review provides evidence to support that culturally enhanced behavioral health interventions for ethnic minority participants are effective [68]. Behavioral theory: As an app of the Social Cognitive Theory construct of behavioral capability, the recipes provide guidance on how to perform a health behavior (healthy eating) [34].

Abbreviations: app, application; CV, cardiovascular; FAITH!, Fostering African-American Improvement in Total Health.

### Education modules

#### Video series

On the basis of feedback from Phase 1, we focused on the development of an interactive platform covering a variety of CV health topics and visuals. A video series comprising 10 education modules for promoting CV health was created as the core app educational tool. The intervention was intended for participants to follow a weekly schedule, concentrated on 1 module each week. To provide evidence-based health information, Mayo Clinic health professionals developed PowerPoint presentation slides for specific module sections, including CV health behaviors and factors (eg, hypertension, diabetes mellitus, healthy eating, physical activity, and stress management). Each module also reinforced messages for changing behaviors to healthful ones. In addition, relevant CV health disparities affecting AAs were also emphasized. The principal investigator and a digital communication and eLearning expert (R.E.S.) reviewed the slides and scripts for concise language and readability (use of bullet points and language at or below a tenth-grade level), cultural appropriateness (language, visuals), and ease of learning/understanding (high-quality diagrams). Next, the experts recorded their presentations in an onsite studio at Mayo Clinic. Each education module included 2 to 5 topic-focused videos of less than 5 minutes.

### Self-assessments

To enhance interactivity, self-guided learning, and retention, we developed brief pre- and post-module assessments, consisting of 3 to 5 multiple choice or true/false questions. The user was required to complete the pre-module assessment in order to unlock the module content (ie, videos and brochures). The post-module assessment provided immediate and informative feedback intended to support and strengthen understanding of the key concepts of each CV health topic.

### Brochure content

We consolidated existing, culturally appropriate, healthy lifestyle materials from reputable governmental agencies (National Heart, Lung, and Blood Institute; Office of Research on Women’s Health; and National Institute for Diabetes, Digestive and Kidney Diseases) [70] and national health organizations (American Heart Association) [71] to complement the education modules, given their high acceptability and user satisfaction among a similar demographic group during our face-to-face program [19]. In particular, we decided to integrate into our modules portions of a culturally appropriate brochure on preventing CV disease from the National Heart, Lung, and Blood Institute called *On the Move for African-Americans*, which is an easy-to-read, digitally formatted educational booklet [72].

### Self-monitoring/tracking

We created a tracking feature for diet (fruit and vegetable intake) and physical activity (daily steps and types of activities) to help engage participants and to challenge them to improve health behaviors. Participants could enter information daily (ie, number of servings, number of steps, minutes, and type of physical activity) using drop-down menus.

### Sharing board

Input from Phase 1 indicated a need for participants to remain connected to the entire group through social networking. Therefore, we included a sharing board with a running feed to allow participants to post messages, videos, and photographs sharing their challenges and successes toward healthy behavior change with the other participants. Other participants were able to like and respond to posts on the feed. Participants had the options of using their own names or an alias for their sharing-board personas. The sharing board was open to all study participants from all participating churches. The study team viewed this feature as a vital means for keeping the participants engaged with the app beyond the education modules.

### Testimonials

Participants of the prior face-to-face FAITH! Program [73] and church leaders recorded video testimonials of their experiences with FAITH! to increase the credibility of the program and relevance to the AA faith community. The testimonials included motivational and spiritual themes to promote healthy lifestyle change.

### Recipes

On the basis of feedback from Phase 1, we learned that participants enjoyed preparing new heart-healthy dishes and meals. Therefore, we created a separate recipe section and included cookbooks with healthy recipes for traditional AA cuisine and Mediterranean and DASH (Dietary Approaches to Stop Hypertension)–style diets [74].

### Phase 3: Iterative prototyping

#### Findings

Sequential, iterative meetings with church partners included detailed review of interactive mock-ups of the semifunctional app from Phase 2. A summary of the feedback received from the group and modifications implemented to enhance the app features are listed in Table 3.

**Table 3:** Phase 3: Church partner FAITH! App prototype feedback (n = 11).

Feedback	App/Intervention Modifications Based on Feedback
**Likes (most useful features):**	
1. Homepage organization by education-module icons	1. Enhanced aesthetic and minimalist design for simple, intuitive use.
2. Succinct video presentations	2. Edited presentations for concise language with minimal medical jargon.
3. Variety of CV health topics	3. Included 10 topics focused on key CV risk factors and psychosocial influences (eg, stress management).
4. Visual depictions of African-Americans	4. Included photographic imagery of African-Americans engaged in health-promoting activities on each app page.
5. Spiritual/biblical messaging	5. Incorporated biblical themes and scriptures to each education module home page.
**Dislikes (suggestions for improvement):**	
1. Unclear learning objectives of education modules	1. Included clearly defined learning objectives at homepage of each module. Also included introduction and summary videos for each module to define the specific CV topic and provide “take-home” points by the study principal investigator for consistent messaging.
2. Small font sizes	2. Provided ability for user to adjust font size in the *Settings* app page.
3. Tracking data entry	3. Added monthly calendar for improved ease of daily entry with drop-down menus for fruit and vegetable intake and physical activity.
4. Intuitiveness/order of sharing board posts	4. Arranged sharing board posts in real-time, reverse chronological order with date and time.
5. Inability to tag others within sharing board post	5. Included function within sharing board to tag participants in messages with use of name “handle.”
6. Lack of speaker biographical information (include section with photographs)	6. Included a brief biographical sketch of each education module video speaker with an accompanying photograph in the *About* app page.
7. Lack of notifications or reminders to engage with the intervention	7. Automated, weekly email reminders were sent to encourage participants to follow the 10-week education module schedule (CV health topic of the week) and to use the other app features (tracking, sharing board).
**Overall impressions:**	
1. Culturally relevant to African-Americans	
2. High-quality graphics and visuals	
3. Easy to navigate	

Abbreviations: app, application; CV, cardiovascular; FAITH!, Fostering African-American Improvement in Total Health.

Meeting 1: Preliminary App Assessment. The input collected from the group informed revisions to the app homepage (font/text size, colors, icons, logo), education modules (defined learning objectives), tracking feature (monthly calendar suggested for user friendliness), and sharing board (clearer directions on how to post messages, tagging option). The group suggested clearly defined learning objectives for each education module, so these were added to each module’s home page. In addition, introduction and summary videos from the principal investigator were included for each module that oriented the user to the weekly CV health topics and speakers and provided key take-home points. The group suggested inclusion of speaker biographical information and photographs to provide background of their particular expertise to the education modules. Notifications and reminders were suggested by the group to enhance engagement and intervention adherence. In response to this feedback and preferences from Phase 1, automatically generated, weekly email reminders were sent to encourage participants to adhere to the 10-week intervention schedule. Although not an inherent feature of the app itself, the reminders highlighted the CV health topic of the week and encouraged participants to use the other app features (tracking, sharing board). The group approved the overall app infrastructure (education modules as the core) and the content of the other features (ie, tracking feature, sharing board, recipes). The church partners and study team agreed to name the app prototype the FAITH! App. The study team then reviewed the meeting summary analysis and revisited the MoSCoW prioritization to rank the importance of suggested changes in the grand scheme of the intervention aims.

Meeting 2: Cultural Context Assessment. The community partners were vocal about the importance of an AA–specific research study within their communities and their personal enjoyment with being a part of a “groundbreaking” effort to promote CV health through an mHealth intervention. One participant stated, “I think the program will provide a way of getting information…uniquely to AAs because we’re different. We need to close that gap and not have such a stigma in our minds as to how research works.”

The community partners also viewed the app as a means to address health disparities by providing a convenient way to increase access to quality health care information among the AA community: “I like the fact that the FAITH! Program is connecting us with experts…through the app to listen to doctors…directly related to a health issue; that’s a big one in our community…because of the disparities….”

Many considered their participation as a part of their church’s health ministry and as role modeling for future generations: “…You can pray all you want to, but there’s certain things you’ve got to do, you know to take care of your body…this temple that God gave us….” Participants appreciated the use of photographs and graphics of AAs, biblical inferences, and scriptures (including a link to a Bible website). These features were viewed as cultural-tailoring efforts of the study team to “meet people where they are.”

The group also noted the multilevel thread of partnerships built by the study (ie, Mayo Clinic and the AA community, Rochester and Minneapolis-St. Paul churches, and FAITH! Partners).

Meeting 3: Final App Prototype Review. The study team made minor revisions to the homepage, as suggested, and all participants approved of the optimized app design, features, and content and its use in the proposed pilot study. Overall, the community partners felt a high level of investment in the success of the FAITH! App and the project as a whole. They expressed how the iterative process allowed their voices to be heard as they were able to see their creative ideas built into an mHealth tool for themselves and others like them: “The app comes together, and it’s like, wow! Your input all matters, and it actually will help in the end and improve not just our health, but others.”

The partners also believed their participation was a way to overcome the traditional authoritative approach of engaging underserved racial/ethnic minority groups within research. They were pleased with being integrated into the CBPR process and felt that they were listened to: “It’s a great example of research done right because a lot of times people will come into a setting being told, you know, this is what you should do or this is the way we’re going to do it vs ask for the feedback, and that gives you, definitely, a more equitable outcome…definitive of the group that you’re trying to get the information from or get the data from for your research. So it’s really exciting for me to see even that whole process…to pull it together.”

### Phase 4: Pilot study

#### Findings

Sociodemographic characteristics of the pilot participants, including patterns of mobile technology use, have been previously reported [33]; in brief, the average age of participants was 49.6 years, most were women (70%), 36% had less than a technical, associate, or college degree, and 49% had an average annual household income <$50,000. The study retention rate was 98% and included participants who completed both baseline and final electronic surveys.

Table 4 summarizes participant perceptions of the FAITH! App categorized by acceptability, usability, and satisfaction outcomes. The intervention was highly acceptable to participants given its high average rating score of 9 (IQR, 8–10; scale, 1 to 10) [56]. Furthermore, participants indicated that overall the FAITH! App reasonably met their needs for gaining knowledge and skills to make healthy lifestyle changes (mean [SD], 3.2 [0.7]; scale, 1 to 4), and they were highly likely to recommend the app to others (mean [SD], 4.5 [0.8]; scale, 1 to 5). The intervention was also considered easy to use according to each subscale of the adapted Health-ITUES and by overall score (mean [SD], 4.4 [0.8]; scale, 1 to 5). User satisfaction overall was high: 93% of participants rated their general satisfaction with the FAITH! App as “satisfied to very satisfied.” Each of the FAITH! App features received a 70% or greater satisfaction rating (satisfied to very satisfied) by over 70% of participants with the exception of the sharing board (64.3%).

**Table 4:** Phase 4 (pilot study): FAITH! App acceptability, usability, and satisfaction^a^.

Measure	Result
Acceptability, mean (SD**)**	
Meets needs for gaining knowledge/skills to make healthy lifestyle changes^b^	3.2 (0.7)
Likelihood of recommending to family member, friend, or colleague^c^	4.5 (0.8)
Overall rating, median (IQR)^d^	9 (8–10)
Usability, mean (SD)^e^	
Impact	4.3 (0.9)
Usefulness	4.6 (1.0)
Perceived ease of use	4.6 (0.8)
User control	4.1 (0.9)
Overall score^c^	4.4 (0.8)
Satisfaction (satisfied/very satisfied), No. (%)^f^	
Education modules	40 (95.2)
Audiovisuals	39 (92.9)
Brochure content	36 (85.7)
Tracking feature	30 (71.4)
Sharing board	27 (64.3)
Testimonials^g^	31 (75.6)
Recipes	37 (88.1)
Overall	39 (92.9)

Abbreviations: app, application; FAITH!, Fostering African-American Improvement in Total Health; IQR, interquartile range; SD, standard deviation.

^a^ Thirty-six participants completed the acceptability and usability outcomes sections of the post-intervention survey, and 42 participants completed the satisfaction outcome section of the final survey.

^b^ Scale, 1 to 4 (not at all well to extremely well).

^c^ Scale, 0 to 5 (not at all likely to extremely likely).

^d^ Scale, 1 to 10 (poor to excellent).

^e^ Adapted from Health Information Technology Usability Evaluation Scale [58]. Nine of 20 subscale items were included from the instrument. The overall usability score was the mean of all the items with each item weighted equally. Scale, 1 to 5 (strongly disagree to strongly agree).

^f^ Scale, dissatisfied to very satisfied.

^g^ Data missing for 1 participant.

## Discussion

We successfully applied a CBPR approach to adapt a highly acceptable but resource-intensive, face-to-face CV disease prevention program to an mHealth lifestyle intervention to increase its accessibility within the AA community. To our knowledge, this is the first community-based mHealth lifestyle intervention to use this formative, comprehensive process in partnership with AA church congregations, with the goal to co-design a digital app focused on improving the CV health of AAs. We engaged the community in all phases of the intervention development process, which allowed us to better understand their mobile technology preferences and experiences. This model resulted in a community-advocated and jointly developed, culturally tailored, CV health and wellness app prototype (FAITH! App). Our subsequent pilot testing of the FAITH! App demonstrated its high acceptability, usability, and satisfaction by an underserved AA community. Furthermore, our emphasis on overcoming CV disease disparities plaguing the AA community through CV health promotion within the education component of the FAITH! App likely further increased its cultural relevance and relatability. Altogether, these attentive strategies were likely contributory to our high retention rate. Our methods can serve as a model of best design practices for other investigators seeking to develop mHealth lifestyle interventions for use by underserved racial/ethnic minority groups. mHealth is a promising, novel method for delivering health promotion and health care messages to improve health outcomes. However, to maximize the uptake potential of mHealth, the needs and preferences of minority groups in using these tools must be understood [75, 76].

Our iterative intervention design process, which was conducted in collaboration with AA community members, allowed us to determine the important features and functions to integrate into the mHealth lifestyle intervention. Although no similar interventions exist that incorporate a digital app specifically for CV health promotion among AAs, 2 CV mHealth lifestyle interventions were also developed recently with a CBPR framework for the AA population and showed feasibility and acceptability [26, 27]. Like these investigators, we see the potential for using these interventions in resource-poor, underserved communities, which unfortunately have the greatest CV health disparities. We learned a number of important lessons throughout our collaborative process and have recommendations that may be of assistance to other researchers developing mHealth interventions: 1) Include the prioritized population throughout the design process to better tailor the intervention to this group; 2) Meticulously outline a sequential timeline of project phases for intervention development and testing to facilitate achievement of set deliverables and milestones; 3) Be flexible to account for community and stakeholder preferences, app developer procedures, and unexpected delays and setbacks; and 4) Incorporate mixed methods into design and pilot testing because this offers rich information to create a well-designed, acceptable intervention for potential end users.

### Strengths and limitations

The major strength of our study was its use of formative research methods for intervention development with multitiered evaluation (iterative community meetings and real-world pilot testing) within the prioritized population. This systematic process directly complemented our use of a CBPR approach with the AA faith community and allowed us to gain deeper insights into their intervention preferences. Our primary collaboration with AA churches provided us with the infrastructure of a trusted institution for health promotion within the community and a practical means to recruit participants and obtain their perspectives [18, 77]. Furthermore, our app is culturally tailored, based on sound theoretical models for behavior change, and consolidates multiple recommended components of effective health-related apps (ie, self-monitoring, performance, and progression) [64]. Lastly, we provide both qualitative and quantitative findings, including detailed app user feedback and ratings.

Our study has several limitations. Inherent in pilot studies, the sample size was relatively small but sufficient for development and beta testing of mHealth interventions [39, 40, 78]. We used a convenience sample which limits the generalizability of our results; however, our study participants were diverse in terms of background, geographic location (small and large metropolitan areas), and socioeconomic status. Most participants were women, but this is an accurate reflection of demographic characteristics of AA churches and the AA faith community [77]. Despite these limitations, our pilot study had a high retention rate, which is reflective of our rigorous intervention design and implementation through a CBPR approach.

Based on our pilot study results, our next step is to examine the efficacy of the FAITH! App in a randomized controlled trial to assess improvements on CV health outcomes.

### Conclusions

This study demonstrated a process that can be used by others for designing new mobile technology–based interventions for behavior change to prevent CV disease among AAs and other underserved communities. Our efforts to integrate formative research and CBPR approaches to intervention design yielded a culturally relevant tool (FAITH! App) aimed at addressing CV health disparities within the AA community.

## Supporting information

S1 File App_design_manuscript_PLOS-rawdata. (CSV)

## Abbreviations

AA: African-American
app: application
CBPR: community-based participatory research
CV: cardiovascular
DASH: Dietary Approaches to Stop Hypertension
FAITH!: Fostering African-American Improvement in Total Health
Health-ITUES: Health Information Technology Usability Evaluation Scale
IQR: interquartile range
mHealth: mobile health
MoSCoW: Must have, Should have, Could have, Would like
SD: standard deviation

## References

[1] Benjamin EJ, Muntner P, Alonso A, Bittencourt MS, Callaway CW, Carson AP, et al Heart Disease and Stroke Statistics-2019 Update: A Report From the American Heart Association. Circulation. 2019;139(10):e56–e66. Epub 2019/02/01. 10.1161/CIR.0000000000000659 . 30700139

[2] National Quality Forum. Disparities in Healthcare and Health Outcomes in Selected Conditions [Internet]. Washington, DC 2017 [cited 2019 Mar 21]. Available from: https://www.qualityforum.org/Publications/2017/01/Disparities_in_Healthcare_and_Health_Outcomes_in_Selected_Conditions.aspx.

[3] American Heart Association. FACTS: Bridging the Gap. CVD and Health Equity [Internet]. Washington, DC; 2012 [cited 2019 Mar 21]. Available from: https://www.heart.org/idc/groups/heart-public/@wcm/@adv/documents/downloadable/ucm_301731.pdf.

[4] Lu Y, Ezzati M, Rimm EB, Hajifathalian K, Ueda P, Danaei G. Sick Populations and Sick Subpopulations: Reducing Disparities in Cardiovascular Disease Between Blacks and Whites in the United States. Circulation. 2016;134(6):472–85. Epub 2016/06/22. 10.1161/CIRCULATIONAHA.115.018102 27324491 PMC5001154

[5] Centers for Disease Control and Prevention, National Center for Chronic Disease Prevention and Health Promotion, Division of Population Health. BRFSS Prevalence & Trends Data [online], 2015. Retrieved from: https://www.cdc.gov/brfss/brfssprevalence/index.html. Accessed February 13, 2018.

[6] Center for Health Statistics, Minnesota Department of Health: Populations of Color in Minnesota. Health Status Report. 2009.

[7] Minnesota Department of Health, 2011. Chronic Diseases and Their Risk Factors in Minnesota: 2011. St. Paul, MN.

[8] Shanedling S, Schardin S. The Minnesota Heart Disease and Stroke Prevention Plan 2011–2020: a progress report. Minn Med. 2013;96(5):44–5. Epub 2013/07/10. . 23833836

[9] Carnethon MR, Pu J, Howard G, Albert MA, Anderson CAM, Bertoni AG, et al Cardiovascular Health in African Americans: A Scientific Statement From the American Heart Association. Circulation. 2017;136(21):e393–e423. 10.1161/CIR.0000000000000534 . 29061565

[10] Homko CJ, Santamore WP, Zamora L, Shirk G, Gaughan J, Cross R, et al Cardiovascular disease knowledge and risk perception among underserved individuals at increased risk of cardiovascular disease. J Cardiovasc Nurs. 2008;23(4):332–7. 10.1097/01.JCN.0000317432.44586.aa . 18596496

[11] Wagner J, Abbott G, Lacey K. Knowledge of heart disease risk among spanish speakers with diabetes: the role of interpreters in the medical encounter. Ethn Dis. 2005;15(4):679–84. . 16259493

[12] Mosca L, Hammond G, Mochari-Greenberger H, Towfighi A, Albert MA, American Heart Association Cardiovascular D, et al Fifteen-year trends in awareness of heart disease in women: results of a 2012 American Heart Association national survey. Circulation. 2013;127(11):1254–63, e1-29. 10.1161/CIR.0b013e318287cf2f 23429926 PMC3684065

[13] Israel BA. Methods in community-based participatory research for health. San Francisco, Calif: Jossey-Bass; 2005.

[14] Schulz AJ, Israel BA, Mentz GB, Bernal C, Caver D, DeMajo R, et al Effectiveness of a walking group intervention to promote physical activity and cardiovascular health in predominantly non-Hispanic black and Hispanic urban neighborhoods: findings from the walk your heart to health intervention. Health Educ Behav. 2015;42(3):380–92. 10.1177/1090198114560015 25819980 PMC4446166

[15] Yeary KH, Cornell CE, Turner J, Moore P, Bursac Z, Prewitt TE, et al Feasibility of an evidence-based weight loss intervention for a faith-based, rural, African American population. Prev Chronic Dis. 2011;8(6):A146 22005639 PMC3221585

[16] Skolarus LE, Zimmerman MA, Bailey S, Dome M, Murphy JB, Kobrossi C, et al Stroke Ready Intervention: Community Engagement to Decrease Prehospital Delay. J Am Heart Assoc. 2016;5(5). 10.1161/JAHA.116.003331 27208000 PMC4889198

[17] Campbell MK, Hudson MA, Resnicow K, Blakeney N, Paxton A, Baskin M. Church-based health promotion interventions: evidence and lessons learned. Annu Rev Public Health. 2007;28:213–34. Epub 2006/12/13. 10.1146/annurev.publhealth.28.021406.144016 . 17155879

[18] DeHaven MJ, Hunter IB, Wilder L, Walton JW, Berry J. Health programs in faith-based organizations: are they effective? Am J Public Health. 2004;94(6):1030–6. Epub 2004/07/14. 10.2105/ajph.94.6.1030 15249311 PMC1448385

[19] Brewer LC, Balls-Berry JE, Dean P, Lackore K, Jenkins S, Hayes SN. Fostering African-American Improvement in Total Health (FAITH!): An Application of the American Heart Association's Life's Simple 7 among Midwestern African-Americans. J Racial Ethn Health Disparities. 2017;4(2):269–81. Epub 2016/04/10. 10.1007/s40615-016-0226-z . 27059054 PMC5516637

[20] Brewer LC, Morrison EJ, Balls-Berry JE, Dean P, Lackore K, Jenkins S, et al Preventing cardiovascular disease: Participant perspectives of the FAITH! Program. J Health Psychol. 2017:1359105317695878. 10.1177/1359105317695878 . 28810418 PMC5957782

[21] WHO Global Observatory for eHealth, & World Health Organization. MHealth: New horizons for health through mobile technologies. Geneva: World Health Organization; 2011 Available from: http://www.who.int/goe/publications/goe_mhealth_web.pdf.

[22] Abebe NA, Capozza KL, Des Jardins TR, Kulick DA, Rein AL, Schachter AA, et al Considerations for community-based mHealth initiatives: insights from three Beacon Communities. J Med Internet Res. 2013;15(10):e221 10.2196/jmir.2803 24128406 PMC3806518

[23] Kelli HM, Witbrodt B, Shah A. The Future of Mobile Health Applications and Devices in Cardiovascular Health. Euro Med J Innov. 2017;2017:92–7. 28191545 PMC5298843

[24] Odulana AA, Kim MM, Isler MR, Green MA, Taylor YJ, Howard DL, et al Examining characteristics of congregation members willing to attend health promotion in African American churches. Health Promot Pract. 2014;15(1):125–33. Epub 2013/03/16. 10.1177/1524839913480799 . 23493800

[25] Burke LE, Ma J, Azar KM, Bennett GG, Peterson ED, Zheng Y, et al Current Science on Consumer Use of Mobile Health for Cardiovascular Disease Prevention: A Scientific Statement From the American Heart Association. Circulation. 2015;132(12):1157–213. 10.1161/CIR.0000000000000232 . 26271892 PMC7313380

[26] Skolarus LE, Cowdery J, Dome M, Bailey S, Baek J, Byrd JB, et al Reach Out Churches: A Community-Based Participatory Research Pilot Trial to Assess the Feasibility of a Mobile Health Technology Intervention to Reduce Blood Pressure Among African Americans. Health Promot Pract. 2017:1524839917710893 10.1177/1524839917710893 . 28583024 PMC6813820

[27] Yingling LR, Brooks AT, Wallen GR, Peters-Lawrence M, McClurkin M, Cooper-McCann R, et al Community Engagement to Optimize the Use of Web-Based and Wearable Technology in a Cardiovascular Health and Needs Assessment Study: A Mixed Methods Approach. JMIR Mhealth Uhealth. 2016;4(2):e38 10.2196/mhealth.4489 27113680 PMC4861844

[28] Henderson VA, Barr KL, An LC, Guajardo C, Newhouse W, Mase R, et al Community-based participatory research and user-centered design in a diabetes medication information and decision tool. Prog Community Health Partnersh. 2013;7(2):171–84. 10.1353/cpr.2013.0024 23793248 PMC4117400

[29] Bennett GG, Steinberg DM, Stoute C, Lanpher M, Lane I, Askew S, et al Electronic health (eHealth) interventions for weight management among racial/ethnic minority adults: a systematic review. Obes Rev. 2014;15 Suppl 4:146–58. 10.1111/obr.12218 . 25196411

[30] Ray R, Sewell AA, Gilbert KL, Roberts JD. Missed Opportunity? Leveraging Mobile Technology to Reduce Racial Health Disparities. J Health Polit Policy Law. 2017;42(5):901–24. 10.1215/03616878-3940477 . 28663182

[31] Maar MA, Yeates K, Perkins N, Boesch L, Hua-Stewart D, Liu P, et al A Framework for the Study of Complex mHealth Interventions in Diverse Cultural Settings. JMIR Mhealth Uhealth. 2017;5(4):e47 10.2196/mhealth.7044 28428165 PMC5418524

[32] Kaushik B, Brunette M, Fu X, Liu B. Customizable, scalable and reliable community-based mobile health interventions 2014.

[33] Brewer LC, Jenkins S, Lackore K, Johnson J, Jones C, Cooper LA, et al mHealth Intervention Promoting Cardiovascular Health Among African-Americans: Recruitment and Baseline Characteristics of a Pilot Study. JMIR research protocols. 2018;7(1):e31 Epub 2018/02/02. 10.2196/resprot.8842 . 29386174 PMC5812978

[34] Bandura A. Social foundations of thought and action: A social cognitive theory. Englewood Cliffs, NJ: Prentice Hall 1986.

[35] Person B, Cotton D. A model of community mobilization for the prevention of HIV in women and infants. Prevention of HIV in Women and Infants Demonstration Projects. Public Health Rep. 1996;111 Suppl 1:89–98. 8862163 PMC1382049

[36] Wallerstein N, Minkler M, Carter-Edwards L, Avila M, Sánchez V. Improving Health Through Community Engagement, Community Organization, and Community Building In: Glanz K, Rimer BK, Viswanath K, editors. Health behavior: theory, research, and practice. Fifth ed San Francisco, CA: Jossey-Bass; 2015 p. 277–300.

[37] Hou S-I, Charlery S-AR, Roberson K. Systematic literature review of Internet interventions across health behaviors. Health Psychology and Behavioral Medicine. 2014;2(1):455–81. 10.1080/21642850.2014.895368 25750795 PMC4345904

[38] Heckman C, Darlow S, Munshi T, Caruso C, Ritterband L, Raivitch S, et al Development of an Internet Intervention to Address Behaviors Associated with Skin Cancer Risk among Young Adults. Internet Interv. 2015;2(3):340–50. 10.1016/j.invent.2015.04.003 26640776 PMC4669098

[39] Hilgart MM, Ritterband LM, Thorndike FP, Kinzie MB. Using instructional design process to improve design and development of Internet interventions. J Med Internet Res. 2012;14(3):e89 10.2196/jmir.1890 22743534 PMC3414906

[40] Ritterband LM, Thorndike FP, Cox DJ, Kovatchev BP, Gonder-Frederick LA. A behavior change model for internet interventions. Ann Behav Med. 2009;38(1):18–27. 10.1007/s12160-009-9133-4 19802647 PMC2878721

[41] Bradbury K, Watts S, Arden-Close E, Yardley L, Lewith G. Developing digital interventions: a methodological guide. Evid Based Complement Alternat Med. 2014;2014:561320 10.1155/2014/561320 24648848 PMC3932254

[42] Neiger BL, Thackeray R. Application of the SMART Model in Two Successful Social Marketing Projects. American Journal of Health Education American Journal of Health Education. 2013;33(5):301–3.

[43] Fetters MD, Curry LA, Creswell JW. Achieving integration in mixed methods designs-principles and practices. Health services research. 2013;48(6 Pt 2):2134–56. Epub 2013/11/28. 10.1111/1475-6773.12117 24279835 PMC4097839

[44] Palinkas LA, Horwitz SM, Green CA, Wisdom JP, Duan N, Hoagwood K. Purposeful Sampling for Qualitative Data Collection and Analysis in Mixed Method Implementation Research. Administration and policy in mental health. 2015;42(5):533–44. Epub 2013/11/07. 10.1007/s10488-013-0528-y 24193818 PMC4012002

[45] Charmaz K. Constructing grounded theory: a practical guide through qualitative analysis. London; Thousand Oaks, Calif: Sage Publications; 2006 xiii, 208 pages p.

[46] Kotarba JA. Symbolic Interaction and Applied Social Research: A FOCUS ON TRANSLATIONAL SCIENCE RESEARCH(1.). Symb Interact. 2014;37(3):412–25. Epub 2014/09/16. 10.1002/symb.111 25221375 PMC4159952

[47] Blumer H. Symbolic interactionism; perspective and method. Englewood Cliffs, N.J.,: Prentice-Hall; 1969 x, 208 pages p.

[48] Iribarren SJ, Cato K, Falzon L, Stone PW. What is the economic evidence for mHealth? A systematic review of economic evaluations of mHealth solutions. PloS one. 2017;12(2). 10.1371/journal.pone.0170581 PMC5289471 28152012

[49] James DC, Harville C 2nd. eHealth Literacy, Online Help-Seeking Behavior, and Willingness to Participate in mHealth Chronic Disease Research Among African Americans, Florida, 2014–2015. Prev Chronic Dis. 2016;13:E156 10.5888/pcd13.160210 27854421 PMC5127175

[50] Maddison R, Pfaeffli L, Whittaker R, Stewart R, Kerr A, Jiang Y, et al A mobile phone intervention increases physical activity in people with cardiovascular disease: Results from the HEART randomized controlled trial. Eur J Prev Cardiol. 2015;22(6):701–9. 10.1177/2047487314535076 . 24817694

[51] Michie S, Yardley L, West R, Patrick K, Greaves F. Developing and Evaluating Digital Interventions to Promote Behavior Change in Health and Health Care: Recommendations Resulting From an International Workshop. J Med Internet Res. 2017;19(6):e232 10.2196/jmir.7126 28663162 PMC5509948

[52] Borrelli B, Ritterband LM. Special issue on eHealth and mHealth: Challenges and future directions for assessment, treatment, and dissemination. Health Psychol. 2015;34S:1205–8. 10.1037/hea0000323 . 26651461

[53] LifeGuide. A Beginners' guide to creating online interventions using LifeGuide 2013 [cited 2017 November 28]. Available from: http://wiki.lifeguideonline.org/w/images/3/39/LifeGuide_Beginners_Guide_14.10.2013.pdf.

[54] Fetters MD, Curry LA, Creswell JW. Achieving integration in mixed methods designs-principles and practices. Health Serv Res. 2013;48(6 Pt 2):2134–56. 10.1111/1475-6773.12117 24279835 PMC4097839

[55] van Dijk JAGM, van Deursen AJAM. Defining Internet Skills. In: Digital Skills: Unlocking the Information Society. New York: Palgrave Macmillan; 2014 p. 21–42.

[56] Schnall R, Cho H, Liu J. Health Information Technology Usability Evaluation Scale (Health-ITUES) for Usability Assessment of Mobile Health Technology: Validation Study. JMIR mHealth and uHealth. 2018;6(1):e4 Epub 2018/01/07. 10.2196/mhealth.8851 . 29305343 PMC5775483

[57] Mobasheri MH, Johnston M, King D, Leff D, Thiruchelvam P, Darzi A. Smartphone breast applications—what's the evidence? Breast. 2014;23(5):683–9. Epub 2014/08/26. 10.1016/j.breast.2014.07.006 . 25153432

[58] Mosca L, Hammond G, Mochari-Greenberger H, Towfighi A, Albert MA. Fifteen-year trends in awareness of heart disease in women: results of a 2012 American Heart Association national survey. Circulation. 2013;127(11):1254–63, e1-29. Epub 2013/02/23. 10.1161/CIR.0b013e318287cf2f 23429926 PMC3684065

[59] Brown CW, Alexander DS, Cummins K, Price AA, Anderson-Booker M. STEPS to a Healthier Heart: Improving Coronary Heart Disease (CHD) Knowledge Among African American Women. American Journal of Health Education. 2018;49(2):57–65. 10.1080/19325037.2017.1414640

[60] Giardina EG, Sciacca RR, Foody JM, D'Onofrio G, Villablanca AC, Leatherwood S, et al The DHHS Office on Women's Health Initiative to Improve Women's Heart Health: focus on knowledge and awareness among women with cardiometabolic risk factors. Journal of women's health. 2011;20(6):893–900. Epub 2011/04/16. 10.1089/jwh.2010.2448 21492002 PMC3113416

[61] Champion V, Skinner C. The health belief model In: Glanz K, Rimer B, Viswanath K, editors. Health Behavior and Health Education: Theory, Research, and Practice. 4th ed San Francisco (CA): Jossey-Bass; 2008 p. 45–65.

[62] Mendiola MF, Kalnicki M, Lindenauer S. Valuable features in mobile health apps for patients and consumers: content analysis of apps and user ratings. JMIR mHealth and uHealth. 2015;3(2):e40 Epub 2015/05/15. 10.2196/mhealth.4283 25972309 PMC4446515

[63] Payne HE, Lister C, West JH, Bernhardt JM. Behavioral functionality of mobile apps in health interventions: a systematic review of the literature. JMIR mHealth and uHealth. 2015;3(1):e20 Epub 2015/03/25. 10.2196/mhealth.3335 25803705 PMC4376122

[64] Higgins JP. Smartphone Applications for Patients' Health and Fitness. Am J Med. 2016;129(1):11–9. 10.1016/j.amjmed.2015.05.038 . 26091764

[65] Riley WT, Rivera DE, Atienza AA, Nilsen W, Allison SM, Mermelstein R. Health behavior models in the age of mobile interventions: are our theories up to the task? Translational behavioral medicine. 2011;1(1):53–71. Epub 2011/07/29. 10.1007/s13142-011-0021-7 21796270 PMC3142960

[66] Dillard AJ, Main JL. Using a health message with a testimonial to motivate colon cancer screening: associations with perceived identification and vividness. Health education & behavior: the official publication of the Society for Public Health Education. 2013;40(6):673–82. Epub 2013/01/29. 10.1177/1090198112473111 . 23355445

[67] Cho YM, Lee S, Islam SMS, Kim SY. Theories Applied to m-Health Interventions for Behavior Change in Low- and Middle-Income Countries: A Systematic Review. Telemedicine journal and e-health: the official journal of the American Telemedicine Association. 2018 Epub 2018/02/14. 10.1089/tmj.2017.0249 . 29437546 PMC6205046

[68] Barrera M Jr., Castro FG, Strycker LA, Toobert DJ. Cultural adaptations of behavioral health interventions: a progress report. Journal of consulting and clinical psychology. 2013;81(2):196–205. Epub 2012/02/01. 10.1037/a0027085 22289132 PMC3965302

[69] Yeager KA, Bauer-Wu S. Cultural humility: essential foundation for clinical researchers. Appl Nurs Res. 2013;26(4):251–6. Epub 2013/08/14. 10.1016/j.apnr.2013.06.008 23938129 PMC3834043

[70] National Heart Lung and Blood Institute, National Institutes of Health. The Heart Truth for African American Women: Take Action to Protect Your Heart [Internet]. 2016 [updated Dec 2016; cited 2019 Mar 21]. Available from: https://www.nhlbi.nih.gov/sites/default/files/publications/16-5066.pdf.

[71] American Heart Association. My Life Check-Life's Simple 7 2018 [cited 2019 Mar 22]. Available from: https://www.heart.org/en/healthy-living/healthy-lifestyle/my-life-check—lifes-simple-7.

[72] National Heart Lung and Blood Institute, National Institutes of Health. On the Move to Better Heart Health for African Americans 2008 [cited 2017 November 28]. Available from: https://www.nhlbi.nih.gov/files/docs/public/heart/aariskfactors.pdf.

[73] Brewer LC, Morrison EJ, Balls-Berry JE, Dean P, Lackore K, Jenkins S, et al Preventing cardiovascular disease: Participant perspectives of the FAITH! Program. J Health Psychol. 2017:1359105317695878 Epub 2017/08/16. 10.1177/1359105317695878 . 28810418 PMC5957782

[74] National Heart Lung and Blood Institute, National Institutes of Health. Heart Healthy Home Cooking African American Style 2008 [cited 2017 November 28]. Available from: https://www.nhlbi.nih.gov/files/docs/public/heart/cooking.pdf.

[75] Stowell E, Lyson MC, Saksono H, Wurth RC, Jimison H, Pavel M, et al. Designing and Evaluating mHealth Interventions for Vulnerable Populations: A Systematic Review. Proceedings of the 2018 CHI Conference on Human Factors in Computing Systems; Montreal QC, Canada. 3173589: ACM; 2018. p. 1–17.

[76] Veinot TC, Mitchell H, Ancker JS. Good intentions are not enough: how informatics interventions can worsen inequality. J Am Med Inform Assoc. 2018;25(8):1080–8. Epub 2018/05/23. 10.1093/jamia/ocy052 . 29788380 PMC7646885

[77] Sahgal N, Smith G. A Religious Portrait of African-Americans 2009 [cited 2017 November 28]. Available from: http://www.pewforum.org/2009/01/30/a-religious-portrait-of-african-americans/.

[78] Bastien JM. Usability testing: a review of some methodological and technical aspects of the method. Int J Med Inform. 2010;79(4):e18–23. 10.1016/j.ijmedinf.2008.12.004 . 19345139

